# Nutrient Distribution Indicated Whole-Tree Harvesting as a Possible Factor Restricting the Sustainable Productivity of a Poplar Plantation System in China

**DOI:** 10.1371/journal.pone.0125303

**Published:** 2015-05-20

**Authors:** Xiaomin Ge, Ye Tian, Luozhong Tang

**Affiliations:** College of Forestry, Nanjing Forestry University, 159 Longpan Road, Nanjing 210037, China; DOE Pacific Northwest National Laboratory, UNITED STATES

## Abstract

We evaluated the biomass and contents of five major macronutrients (N, P, K, Ca and Mg) in 10-year-old poplar trees (*Populus deltoids* Bartr. cv. “Lux”), and determined their nutrient use efficiencies (NUEs) at Zhoushan Forestry Farm (32°20′ N, 119°40′ E), Jiangsu province, in eastern China. The above- and below-ground biomass of poplar trees was 161.7 t ha^-1^, of which 53.3% was stemwood. The nutrient contents in the aboveground part were as follows: 415.1 kg N ha^-1^, 29.7 kg P ha^-1^, 352.0 kg K ha^-1^, 1083.0 kg Ca ha^-1^, and 89.8 kg Mg ha^-1^. The highest nutrient contents were in stembark, followed by branches, roots, stemwood, and foliage. The NUEs of the aboveground parts of poplar for N, P, K, Ca and Mg were 0.313, 4.377, 0.369, 0.120, 1.448 t dry biomass kg^-1^ nutrient, respectively, while those of stemwood were 1.294, 33.154, 1.253, 0.667, and 3.328 t dry biomass kg^-1^, respectively. The cycling coefficients, defined as the percentage of annual nutrient return in annual nutrient uptake, of N, P, K, Ca and Mg for the aboveground part were 87, 95, 69, 92, and 84%, respectively. Based on the NUE and nutrient cycling characteristics, shifting from whole-tree harvesting to stemwood-only harvesting and appropriately extending the harvest rotation could prevent site deterioration and support sustainable productivity of poplar plantation systems.

## Introduction

Forests are receiving more attention as resource deficiencies and environmental deterioration become increasingly serious problems worldwide. In 1998, the Chinese Government launched the “Natural Forest Protection Program”, which banned logging and protected natural forests. However, with rapid economic development in recent years, there is an increasing demand for timber. Consequently, since 2002 there have been efforts to establish high-yield plantations of fast-growing tree species. Such plantations are generally managed under short rotations with whole-tree harvesting. The soil becomes degraded after several rotations, increasing concerns about the sustainable productivity of fast-growing plantations [1–4].

Poplar (*Populus spp*.) is a fast-growing genus with high timber yields. Poplar plantations are widely distributed in northern and central China, and play roles in providing wood resources and maintaining ecological stability. In 2007, poplar plantations covered approximately 7 million ha in China [5]. Some researchers have suggested, however, that short-term harvest rotation and whole-tree harvesting of poplar should be the key causations leading to soil degradation and decreased productivity [6–8]. These problems have also been reported for other plantation species, including Chinese fir (*Cunninghamia lanceolata*), masson pine (*Pinus massoniana*), and *Eucalyptus*. Declining soil fertility and plant productivity have been attributed to nutrient deficiency, soil poisoning, soil acidification, and allelopathy [9, 10]. For fast-growing tree species with short rotations and whole-tree harvesting, soil nutrient deficiency is considered to be the main cause of declining productivity [9, 11]. Nutrient characteristics of the removed tree parts also partly contribute to the nutrient costs of biomass production [12].

Nutrient use-efficiency (NUE) is a useful indicator for differentiating the nutrient costs of biomass production among different plantation types [13, 14]. The NUE is related to several factors, including tree species, stand density, soil nutrient reserves, silvicultural manipulation, and harvest rotation [13, 14]. In this study, we hypothesized that nutrient imbalance caused by poor management strategies such as short rotations and whole-tree harvesting, and the characteristics of poplar NUE, are factors in the declining soil fertility and stand productivity in poplar plantations.

In this study, we investigated tree growth and nutrient characteristics of poplars in a 10-year-old stand. The species was *Populus deltoides* Bartr. cv. “Lux”, a widely-used poplar clone that was introduced from North America in the 1970s. The specific objectives were (1) to quantify biomass and nutrient contents in 10-year-old poplar trees, and (2) to evaluate the NUE characteristics of poplar trees in the plantation.

## Materials and Methods

### Ethics statement

Our study did not include humans or animals during the experiment. The Gaoyou City Forestry Bureau issued the permission for our study site. We confirm that the field studies did not involve endangered or protected species. Sampling procedures and experimental manipulations were approved as part of obtaining the field permit.

### Study site

The study was carried out at Zhoushan Forestry Farm (32°20′ N, 119°40′ E), located in Gaoyou City, Jiangsu Province, in eastern China. The Forestry Farm belongs to Gaoyou City Forestry Bureau. This area is in the Lixiahe low-lying wetland region. The average elevation is approximately 5 m above sea level and the mean annual groundwater level is approximately −70 to −80 cm. During the growing season (from April to October), the mean groundwater level was approximately −50 cm. This region has a monsoon sub-humid climate, with a mean annual air temperature of 14.5°C and a mean annual rainfall of approximately 1,000 mm. The soil is derived from lacustrine sediments, and the mineral soil of this area is with a clay texture. The main properties of the soil in the poplar plantation are shown in Table 1 (see soil analysis methods below). This region is suitable for the cultivation of *P*. *deltoides* [5, 15].

**Table 1:** Properties of soil at the study site: a *P*. *deltoides* plantation at the Zhoushan Forestry Farm, Jiangsu, China.

Depth	Bulk density	pH (H_2_O)	Organic C	Total N	Total P	Total K	Total Ca	Total Mg	Exch. K^a^	Exch. Ca^a^	Exch. Mg^a^
(cm)	(g cm^-3^)		(g kg^-1^)								
0–10	1.26 ±0.03	7.1 ±0.1	13.1 ±1.4	1.11 ±0.13	0.43 ±0.05	5.6 ±0.6	5.2 ±0.3	4.4 ±0.2	0.16 ±0.04	3.25 ±0.26	0.43 ±0.04
10–20	1.35 ±0.06	7.1 ±0.1	9.6 ±1.5	0.92 ±0.14	0.33 ±0.08	5.9 ±0.6	4.4 ±0.2	4.3 ±0.3	0.10 ±0.01	2.12 ±0.25	0.46 ±0.01
20–50	1.28 ±0.06	7.2 ±0.1	8.0 ±0.8	0.89 ±0.08	0.29 ±0.04	8.9 ±0.3	5.0 ±0.2	5.8 ±0.2	0.12 ±0.01	3.73 ±0.18	0.62 ±0.08

^a^ Exch., exchangeable. Values shown are mean ± standard error (SE).

The plantation was established in the spring of 2002 using 1-year-old rooted cuttings of *P*. *deltoides* Bartr. cv. “Lux” with uniform height and diameter. The cuttings were planted in the pits with the length, width and depth were about 80 cm, respectively, after cleaning up weeds and tilling soil in the planting area. The planting density was 1,111 stems per ha. By 2010, more than 95% of the trees were conserved. Before the plantation establishment, this site was uncultivated. For the first five years after afforestation, oilseed rape (*Brassica napus*) was planted in the plantation and 375 kg ha^-1^ of compound fertilizer (N: P: K = 15%: 6.55%: 12.45%) (Nanjing chemical industry co., LTD, China) was applied for oilseed rape each year. After 5 years, the stand crown was closed, and no other management was applied in the plantation. For the 10-year-old poplar plantation by 2010 in our study site, the average height and diameter at breast height (DBH) of trees were 19.8 (± 0.4) m and 18.2 (± 0.6) cm, respectively, and the mean basal area and volume were 28.0 m^2^ ha^-1^ and 193.1 m^3^ ha^-1^, respectively. The understory vegetation of the plantation was dominated by *Erigeron annuus*, *Vicia sepium*, *Rubus parvifolius*, and *Morus alba*.

### Sampling and analysis

We established three 500-m^2^ plots at random points in the plantation in October 2010. In October 2010, 2011, and 2012, we measured the DBH and height of all trees in each plot. The biomass of leaves, branches, stems, and roots of each tree were calculated for each year using the following logarithmic equations [16]:

$$\text{lg}\;W_{L}=0.4489\;\text{lg}\;(DBH^{2}\;H)-1.1455$$ (1)

$$\text{lg}\;W_{B}=0.9911\;\text{lg}\;(DBH^{2}\;H)-2.3791$$ (2)

$$\text{lg}\;W_{S}=1.0659\;\text{lg}\;(DBH^{2}\;H)-2.1305$$ (3)

$$\text{lg}\;W_{R}=0.7061\;\text{lg}\;(DBH^{2}\;H)-1.2588$$ (4)

where: *W* is the biomass of different tree tissues (kg), *DBH* is the diameter at breast height of tree (cm), *H* is the height of tree (m), and *L*, *B*, *S*, and *R* are the leaf, branch, stem, and root of poplar, respectively.

Because the plantation was established on flatlands using same poplar clone with uniform rooted cuttings size, and was under same managements, tree height and DBH showed very small variations only within each plot. In addition, it was especially tough job to excavate the whole root system for root classification and sampling. Therefore, in order to reduce field load, and also considering the uniform condition for all the trees in our study site, we selected only one ‘average’ tree from each plot according to average DBH and height for stem analysis. Because poplar is a deciduous tree species, we cut down the average trees and sampled in July, 2011, so that we could collect leave samples. The tree boles were sectioned at the stump, at breast height, and at 2-m intervals above breast height. A disk approximately 5-cm thick was cut from the stump and from the base of each stem section for analysis. The volume of each section was calculated using Huber formula, except that the top section (from the top section to the tip of the tree) was calculated as a geometric cone [17]. Total growth increment, mean annual increment, and current annual increment of mean height, DBH, and volume of a single tree were measured using stem analysis [18, 19]. Each disk was divided into bark and wood to determine the bark-to-wood biomass ratio [20], and then the bark and wood samples were used for nutrient analysis. The mean values of nutrient concentrations in stemwood and stembark for the whole tree were weighted according to the corresponding biomasses of each stem section determined in the stem analysis. All branches were cut from the average trees, and 10 branches with foliage from each sample tree were collected randomly. The foliage of the collected branches were picked and mixed by each sample tree for nutrient analysis. The branches from each sample tree were divided into different age classes (current branch, 2-year-old branch, 3-year-old branch, >3-year-old branch). The mean values of nutrient concentrations in branches were weighted according to the corresponding biomasses of different age classes of branches of each sample tree.

According to the tree spacing, the whole roots of the mean tree in each plot were estimated by excavating and harvesting in a 3×3 m square centered with the mean tree by hand as carefully as possible. Then the root samples were divided into fine roots (Ø < 2 mm) and coarse roots (Ø > 2 mm). We measured the fresh weights of the fine and coarse roots after rinsing dirt, and then measured the moisture content. The total root biomass estimated using Eq (4) was then divided into fine and coarse root biomass according to the fine-to-coarse root biomass ratio. The fine and coarse root samples were also used for nutrient analysis.

In each plot, five 2 × 2 m subplots were selected to sample understory vegetation, and another five 0.5 × 0.5 m subplots were selected to sample the forest floor, in July of 2011. Five litter traps with an internal diameter of 1 m were randomly placed in each plot to collect aboveground litterfall of trees. The litterfall was collected from each litter trap and the biomass and chemical properties were determined each month from October 2010 to October 2011.

After collection, all the plant samples were transported to the laboratory and oven-dried at 65°C to constant weight for biomass determination, and then ground and sieved through a 0.25-mm mesh sieve before nutrient analysis.

Three soil pits were randomly located and then excavated in each plot in October 2010. For poplar roots, especially for fine roots are mostly distributed in 0–50 cm soil layer in our study site, so a soil depth of approximately 50 cm were sampled and analyzed. Soil cores were collected by volumetric rings (100 cm^3^) from the 0–10, 10–20, and 20–50 cm soil layers, and then oven-dried to constant weight. The bulk density of soil was analyzed after removing gravel (>2 mm in diameter) and large pieces of plant residue. Additional soil cores were collected from each soil layer. After air-drying, the soil samples were ground and sieved through a 2-mm mesh sieve before determinations of pH and exchangeable K^+^, Ca^2+^, and Mg^2+^, or sieved through a 0.25-mm mesh sieve before determinations of total N, P, K, Ca, and Mg.

Soil pH was measured using a pH meter at a 1:2.5 (*w/v*) soil-to-water ratio. The plant and soil samples were Kjeldahl-digested with concentrated perchloric and sulfuric acid for total nutrient measurements. Nitrogen (N) and phosphorus (P) concentrations were determined using a flow injection system (Bran+Luebbe AA3, Germany), and potassium (K), calcium (Ca), and magnesium (Mg) concentrations were determined by atomic absorption spectrophotometry (Solaar Unicam 969 AAS, USA). Soil exchangeable K^+^, Ca^2+^, and Mg^2+^ were extracted with 1 M ammonium acetate (pH 7) and then analyzed by atomic absorption spectrophotometry. All chemical analyses were carried out in triplicate.

### Data analysis

The productivity of the 10-year-old poplar plantation was evaluated based on biomass of the plantation in years 9 and 11 following the method described in [21]. Briefly, based on the DBH and height of trees measured in October of 2010 and 2012, the annual biomass increment of the stand was estimated by summing the mean annual biomass changes of individual tissues from 2010 to 2012 using the logarithmic Eq (1)–(4) listed in above. Some indexes for assessing nutrient cycling and NUEs [22,23] in the stands were defined and calculated as follows:

$$\begin{array}{l} \text{Annual nutrient increment}\;=\sum (\text{annual biomass increment of each poplar tissue}\\ \;\times \text{nutrient concentration in each tissue}) \end{array}$$ (5)

$$\begin{array}{l} \text{Annual nutrient return}\;=\sum (\text{aboveground litterfall biomass of poplar each month in the stand}\\ \;\times \text{nutrient concentration in litterfall each month}) \end{array}$$ (6)

Annual nutrient uptake=annual nutrient increment+annual nutrient return (7)

Cycling coefficient(%)=(annual nutrient return/annual nutrient uptake)×100% (8)

NUE1=aboveground biomass of poplar/nutrient content in aboveground part of tree (9)

NUE2=stemwood biomass of poplar/nutrient content of poplar stemwood (10)

The calculations of the average values and the standard errors of the nutrient concentrations in different components of poplar trees, understory vegetations, forest floors, and soils were carried out using Excel (version 2003, Microsoft).

## Results

### Growth and timber volume

**Fig 1:**
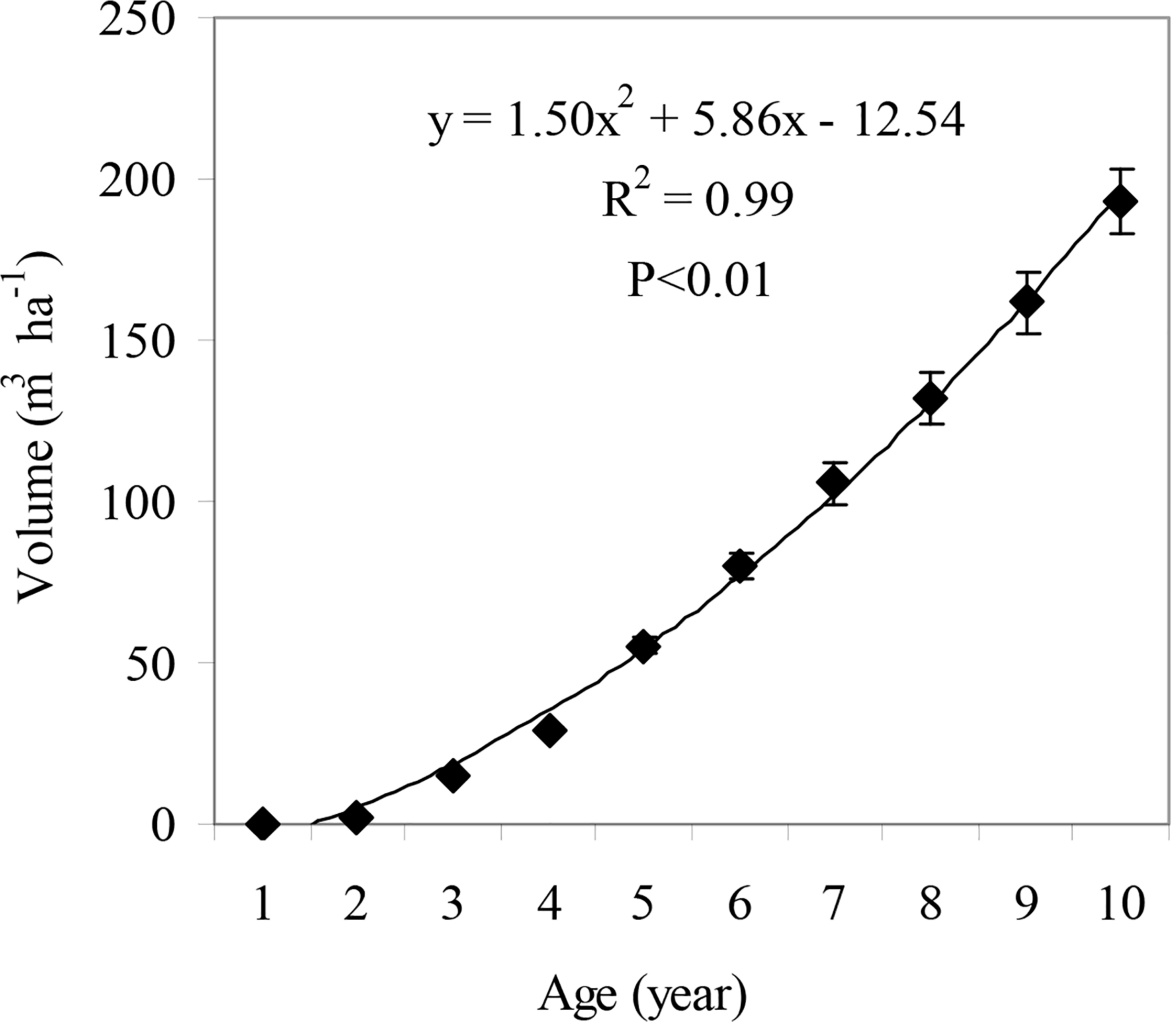
Relationship between stand volume and stand age in *P*. *deltoides* plantation in Zhoushan Forestry Farm, Jiangsu, China. Error bars represent standard error (SE).

**Fig 2:**
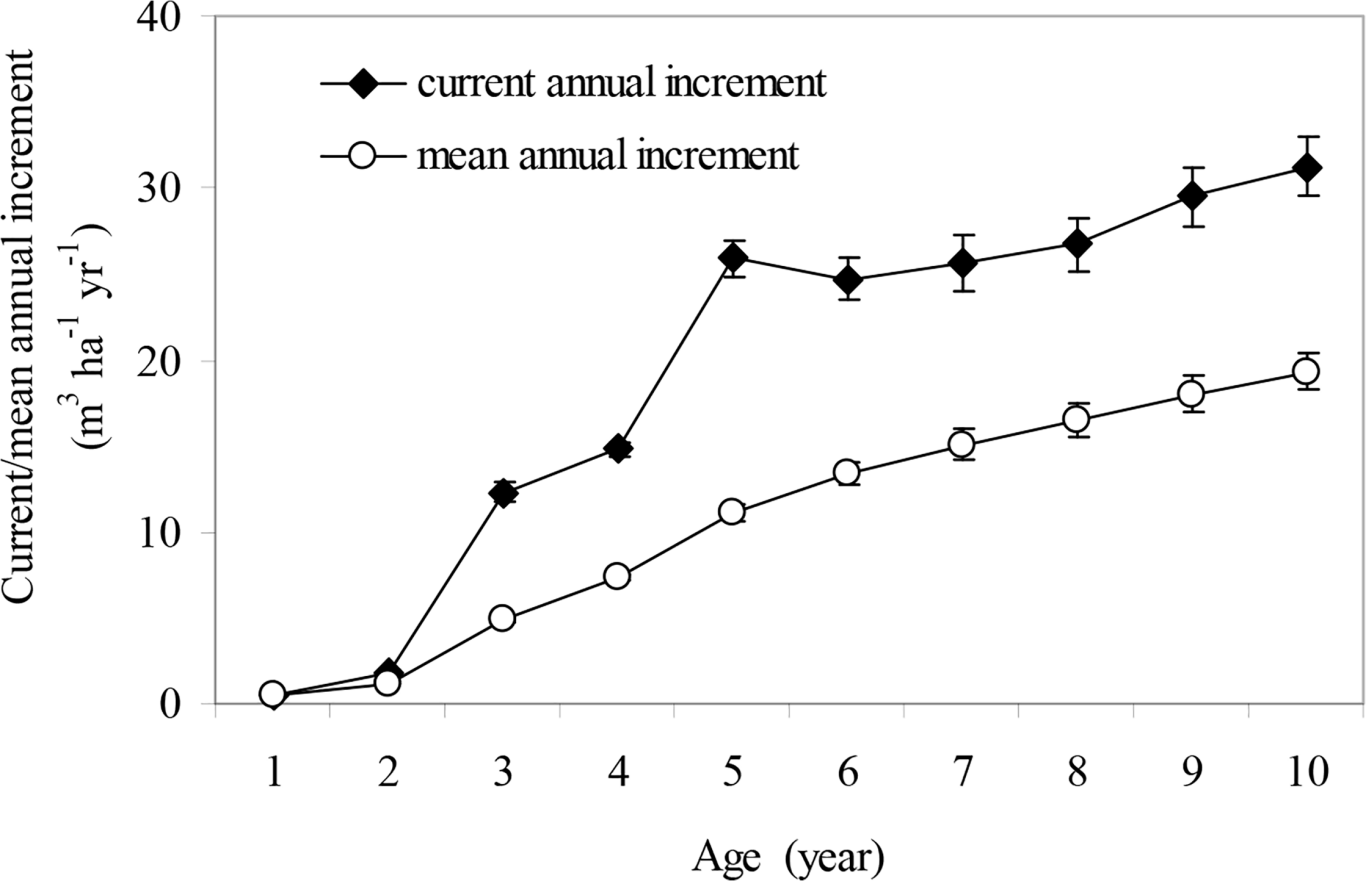
Current annual increment and mean annual increment of stand volume of *P*. *deltoides* plantation at Zhoushan Forestry Farm, Jiangsu, China. Error bars represent standard error (SE).

The stand volume in the poplar plantation increased as the stand aged. There was a quadratic correlation between stand volume and stand age (Fig 1). The stand volume in the 10-year-old plantation was 193.1 m^3^ ha^-1^. Both mean annual increment and current annual increment of the stand volume increased with stand age (Fig 2). In the first 5 years, the current annual volume increment increased rapidly, but slowed over the subsequent 5 years. In contrast, mean annual volume increment increased relatively steadily over the entire 10-year period. The current annual volume increment and mean annual volume increment were 31.3 and 19.3 m^3^ ha^-1^ yr^-1^, respectively. These increments had not yet intersected in the 10-year-old poplar plantation (Fig 2), suggesting that the plantation was not quantitatively mature.

### Nutrient concentrations

We analyzed the nutrient concentrations in the different vegetation components of the poplar plantation (Table 2). Among all of the components, stemwood showed lower nutrient concentrations, while foliage and understory vegetation showed the highest concentrations of N and K. The highest P concentration was in understory vegetation, followed by aboveground litterfall, foliage, forest floor, fine roots, branches, stembark, coarse roots, and then stemwood. However, the Ca concentration was higher in aboveground litterfall, forest floor, stembark, foliage, and fine roots than in the other components. The concentration of Mg was higher in foliage and moderate in forest floor, aboveground litterfall, and understory vegetation, compared with its concentration in the other components. Of the five macronutrients, Ca showed the highest concentration and P the lowest in the components of the poplar plantation.

**Table 2:** Concentrations of selected nutrients in different components of the 10-year-old *P*. *deltoides* plantation ecosystem.

Component	N	P	K	Ca	Mg
	(g kg^-1^)				
Trees					
Foliage	18.65 ± 1.22	1.22 ± 0.06	11.27 ± 0.47	30.58 ± 2.17	4.45 ± 0.06
Living branches	6.58 ± 0.23	0.60 ± 0.01	4.98 ± 0.35	14.17 ± 0.55	0.89 ± 0.06
Stemwood	0.77 ± 0.05	0.03 ± 0.01	0.79 ± 0.06	1.50 ± 0.03	0.30 ± 0.01
Stembark	7.49 ± 0.37	0.51 ± 0.04	7.50 ± 0.17	30.71 ± 0.18	1.59 ± 0.06
Fine roots (Ø <2 mm)	6.91 ± 0.26	0.83 ± 0.09	7.38 ± 0.18	30.29 ± 2.06	1.58 ± 0.36
Coarse roots (Ø >2 mm)	1.89 ± 0.13	0.21 ± 0.03	3.47 ± 0.20	7.67 ± 0.27	0.61 ± 0.03
Understory vegetation	17.14 ± 2.38	2.16 ± 0.32	12.48 ± 1.28	20.12 ± 1.61	3.28 ± 0.26
Aboveground litterfall	11.92 ± 0.31	1.27 ± 0.10	4.31 ± 0.15	38.29 ± 1.47	4.10 ± 0.20
Forest floor	16.28 ± 1.13	1.11 ± 0.02	4.08 ± 0.32	38.18 ± 2.03	4.12 ± 0.12

Values shown are mean ± standard error (SE).

### Biomass and nutrient contents

The estimated biomass and the five tested macronutrient contents in the 10-year-old poplar plantation are shown in Table 3. Total tree biomass was 161.7 t ha^-1^. This accounted for more than 99% of total vegetation biomass (trees + understory vegetation) in the plantation. Among all the tested components, stemwood was the largest, accounting for 53.3% of the total tree biomass. The next largest components were roots, branches, stembark, and foliage. The understory vegetation accounted for only 0.4% of total biomass in the plantation ecosystem. The biomass of the forest floor was 4.7 t ha^-1^.

**Table 3:** Biomass and macronutrient contents in various plant components and soil in the 10-year-old *P*. *deltoides* plantation ecosystem.

Component	Biomass (t ha^-1^)	N	P	K	Ca	Mg
		(kg ha^-1^)				
Trees						
Foliage	3.7 ± 0.3	69.0 ± 3.7	4.5 ± 0.3	41.7 ± 1.9	113.1 ± 9.9	16.5 ± 0.5
Living branches	23.8 ± 1.8	156.6 ± 10.5	14.2 ± 1.2	118.6 ± 4.8	337.2 ± 12.4	21.4 ± 0.9
Stemwood	86.2 ± 3.1	66.6 ± 2.7	2.6 ± 0.2	68.8 ± 3.4	129.2 ± 4.4	25.9 ± 1.3
Stembark	16.4 ± 1.2	122.9 ± 3.8	8.4 ± 1.3	122.9 ± 6.1	503.5 ± 12.1	26.0 ± 2.2
Aboveground	130.0 ± 8.4	415.1 ± 28.1	29.7 ± 2.5	352.0 ± 11.1	1083.0 ± 50.5	89.8 ± 4.2
Belowground	31.7 ± 3.1	61.1 ± 4.8	6.8 ± 0.9	111.5 ± 10.8	245.2 ± 7.6	18.7 ± 0.7
Sum	161.7 ± 12.5	476.2 ± 36.5	36.5 ± 2.3	463.5 ± 21.0	1328.2 ± 47.3	108.5 ± 4.3
Understory vegetation	0.7 ± 0.2	12.0 ± 2.0	1.5 ± 0.2	8.7 ± 1.5	14.1 ± 1.7	2.3 ± 0.1
Forest floor	4.7 ± 0.2	76.6 ± 5.2	5.2 ± 0.2	19.2 ± 2.0	179.4 ± 5.9	19.4 ± 1.0
Soil (0–50 cm)	–	6085 ± 241	2108 ± 283	49117 ± 4634	31758 ± 2183	33506 ± 2610

Values shown are mean ± standard error (SE).

The data indicated that less N, P, K, Ca and Mg were stored in stemwood and foliage than in branches, stembark, or roots. Nutrients accumulated in the stemwood accounted for only 12.2% of nutrients accumulated in the entire tree. The forest floor and understory vegetation showed high nutrient contents relative to their biomass, accounting for 10.9 and 1.4% of total nutrient contents in the entire vegetation system, respectively. The trends for different nutrients accumulated in various components of the plantation were similar to those of nutrient concentrations. Of the nutrients tested, Ca was the most abundant nutrient in each of the tested components, ranging from 36.5% (understory vegetation) to 64.2% (stembark). In the plantation system, more than 97% of the nutrients were stored in soil. Total K, Ca, and Mg accounted for 40.1, 25.9 and 27.3%, respectively, of the total soil nutrient content in the top 50-cm soil layer, while the total N and P contents in soil were relatively low.

### Aboveground nutrient cycling

We determined the annual nutrient uptake, increment, return, and cycling coefficients for each tested macronutrient in the plantation (Table 4). Of the total annual nutrient uptake of 347.4 kg ha^-1^ yr^-1^ in the aboveground part of poplar in the plantation, about 41.4 kg ha^-1^ yr^-1^ was reserved in the poplar trees, while about 306 kg ha^-1^ yr^-1^ returned to the surface layer via aboveground litterfall. The stocks of different nutrients in annual nutrient uptake, increment, and return, all showed similar trends; that is, Ca > N or K > Mg > P. For example, Ca accounted for 61.3% of annual nutrient uptake, 43.5% of annual nutrient increment, and 63.7% of annual nutrient return. Of all the nutrients tested, P accounted for the smallest proportions of annual nutrient uptake, annual nutrient increment, and annual nutrient return. However, P showed the highest nutrient cycling coefficient (as high as 95%), followed by Ca, N, Mg and K. Potassium had the lowest nutrient cycling coefficient of 69%.

**Table 4:** Nutrient cycling of aboveground poplar and Chinese fir at different maturity stages.

Stand	Mature Status	Nutreint cycling parameter	N	P	K	Ca	Mg
Poplar	Near-mature	Annual nutrient uptake (kg ha^-1^ yr^-1^)^a^	70	7.4	32	213	25
		Annual nutrient increment (kg ha^-1^ yr^-1^)^b^	9	0.4	10	18	4
		Annual nutrient return (kg ha^-1^ yr^-1^)^c^	61	7	22	195	21
		Cycling coefficient (%)^d^	87	95	69	92	84
Chinese fir^e^	Mid-aged	Annual nutrient uptake (kg ha^-1^ yr^-1^)	78	15	54	43	10
		Cycling coefficient (%)	39	21	9	44	37
	Mature	Annual nutrient uptake (kg ha^-1^ yr^-1^)	78	15	40	55	15
		Cycling coefficient (%)	44	26	18	58	52

^a^Annual nutrient increment + annual nutrient return

^b^∑ (annual biomass increment of each poplar tissue × nutrient concentration of each tissue)

^c^∑ (aboveground litterfall biomass of poplar of each month in the stand × nutrient concentration in litterfall of each month)

^d^(annual nutrient return / annual nutrient uptake) ×100%; ^e^ data are from Sheng and Fan (2005).

### Nutrient use efficiency (NUE)

The NUEs in different plantation ecosystems are shown in Table 5. Compared with the other main plantation tree species in southern China, poplar in the 10-year-old plantation showed a lower NUE1 for N. Poplar showed the second highest NUE2 values for N and P (highest values were in *Pinus massoniana*), and K (highest value was in *Acacia mangium*). The NUE1 and NUE2 values for Ca and Mg in poplar were lower among values obtained for the main plantation tree species in southern China.

**Table 5:** Nutrient use efficiency of poplar and several plantation tree species in southern China.

Stand	Mature status ^a^	NUE1^b^ (t kg^-1^)					NUE2^c^ (t kg^-1^)					References
		N	P	K	Ca	Mg	N	P	K	Ca	Mg	
Poplar	Near-mature	0.31	4.38	0.37	0.12	1.45	1.29	33.15	1.25	0.67	3.33	This study
Chinese fir	Mid-aged	0.35	1.38	0.30	0.48	1.65	0.81	3.91	0.76	0.78	2.43	[23]
	Mature	0.46	1.24	0.52	0.46	2.64	0.69	1.59	0.89	0.56	5.32	[23]
*Eucalyptus*	Mature	0.57	3.83	0.28	0.27	3.34	1.03	5.26	0.38	0.66	8.33	[24]
*Pinus massoniana*	Mature	0.30	3.01	0.24	0.85	1.06	1.72	100.19	0.99	1.52	0.95	[25]
*Acacia mangium*	Near-mature	0.14	5.73	0.71	0.43	4.06	0.31	20.01	2.17	1.25	16.70	[26]

^a^ Cutting age of plantations in China are as follows: poplar, 12–15 years; Chinese fir, 25–30 years; *Eucalyptus*, 5–10 years; *Pinus massoniana*, 30–40 years; *Acacia mangium*, 10–15 years.

^b^ NUE1 = aboveground biomass of trees (t)/nutrient content (kg) in aboveground parts of trees.

^c^ NUE2 = stemwood biomass of trees (t)/nutrient content (kg) in tree stemwood.

## Discussion

Because of the timber shortages in China, poplar plantations are generally managed as whole-tree harvesting. Our results showed that the concentrations of N, P, K, Ca, and Mg were lower in stemwood than in other parts of poplar trees (Table 2). Stemwood accounts for approximately 66.3% of the aboveground poplar biomass (Table 3), and predominates in the poplar plantation. The nutrient contents in the aboveground part of the poplars were as follows: 415.1 kg N ha^-1^, 29.7 kg P ha^-1^, 352.0 kg K ha^-1^, 1083.0 kg Ca ha^-1^, and 89.8 kg Mg ha^-1^. The nutrient contents in poplar stemwood were much lower: 66.6 kg N ha^-1^, 2.6 kg P ha^-1^, 68.8 kg K ha^-1^, 129.2 kg Ca ha^-1^, and 25.9 kg Mg ha^-1^. Therefore, the amount of macronutrients in stemwood occupied only approximately 14.9% of the total amount of macronutrients in the aboveground part of poplar trees. The distribution of biomass and nutrient in the various tree components of poplar was similar to other main plantation tree species in the world. They all showed that tree crown (mainly includes foliage and branches), even though which represented low biomass constitution, contains large quantities of nutrient accumulation, especially for N and Ca [32–33]. Our results agreed with several other reports, which all indicated that the nutrient concentrations were higher in foliage and branches than in stemwood [29–31], and much of the biomass was in non-stemwood parts of the trees [12, 20]. Therefore, whole-tree harvesting would result in excess removal of nutrients from the poplar plantation ecosystem, and leading to soil degradation [12, 27–29].

The results of this study show that N, K and especially Ca were the nutrients most likely to be lost via whole-tree harvesting. It agreed with previous reports [28]. Additionally, the contents of Ca were the highest in each tissue of poplar, it may be because Ca plays important roles in many physiological processes in plants, and *P*. *deltoides* is a Ca-accumulating tree species [34].

As well as the harvesting method, the developmental stage of the stand also affects the magnitude of nutrient removal [29]. The proportion of nutrient-rich tissues in trees decreases with stand age; accordingly, there are decreases in the amount of nutrients per unit stemwood and total biomass [35–37]. Based on our results, the 10-year-old *P*. *deltoides* plantation was not yet at an optimal stage for harvest (Fig 2) because that the stand volume was still increasing rapidly at age 10 (Fig 1). The results of this study indicate that when harvesting the plantation, if only the stemwood is removed and all the other nutrient-rich parts of the tree are left *in situ*, and if the harvest rotation is extended appropriately, then nutrient losses from the site could be greatly reduced. Furthermore, the harvest residues could also serve as a good shelter to protect the soil from erosion and nutrient leaching [38].

The amounts of nutrients removed from sites generally vary according to biomass yield, and also according to the NUE of the tree species [39]. Generally, nutrient-efficient tree species export low amount of nutrients in harvested stemwood biomass [22]. When expressing NUE1 as metric tons of aboveground biomass per kilogram nutrient content in the aboveground part of trees, the value of NUE1 in poplar for N was lower than those reports of other major plantation tree species in southern China (Table 5). This finding suggested that more N was required to produce same amount of aboveground biomass for poplar than for other tree species. In contrast, the values of NUE2, expressed as metric tons of biomass per kilogram nutrient content in stemwood, for N, P and K for poplar were higher than the corresponding values for other plantation tree species (Table 5). This result suggests that larger amounts of stemwood can be produced with lower nutrient costs in poplar plantations than in plantations of other tree species. The small NUE1 and NUE2 values for both Ca and Mg in this 10-year-old poplar plantation may be related to the high availability of exchangeable Ca^2+^ and Mg ^2+^ at this study site (Table 1) [13]. The other main plantation tree species in southern China also showed higher NUE in stemwood compared to the whole tree (Table 5). These findings confirm the importance of stemwood-only harvesting to leave the nutrient-rich parts of trees on site [22].

There should be a relationship between nutrient cycling and sustainable production in plantation systems [34]. In this study, annual nutrient uptake of the 10-year-old poplar plantation was estimated to be 347.4 kg ha^-1^ yr^-1^ (total of N, P, K, Ca, and Mg); of which, 88% was returned to soil via aboveground litterfall. In a forest ecosystem, nutrient input in soil generally includes aboveground litterfall, fine root turnover, canopy leaching, deposition and weathering [40–46]. In the present study, only aboveground litterfall from trees was measured. Therefore, the estimated annual nutrient return and cycling coefficients should be less than the actual values. In a review paper, Block et al. [47] reported that fine root turnover rate of *Populus* spp. range from 0.5 yr^-1^ to 1.8 yr^-1^ in older poplar plantations. According to some other studies of poplar with stand ages similar to our study stand, fine root turnover rate was between 0.86 yr^-1^ and 1.32 yr^-1^ [48–50]. Using the latter report rates, the annual nutrient return from fine root turnover in our study site was estimated to be 37.5–43.4 kg N ha^-1^ yr^-1^, 4.5–5.2 kg P ha^-1^ yr^-1^, 40.0–46.3 kg K ha^-1^ yr^-1^, 164.2–190.1 kg Ca ha^-1^ yr^-1^, and 8.6–9.9 kg Mg ha^-1^ yr^-1^. Deposition from atmospheric precipitation, especially for nitrogen, is another important input into plantation ecosystems. Nitrogen deposition has been reported more than 25 kg N ha^-1^ yr^-1^ in many regions in China, especially in heavy industrial pollution areas [51–52], which was significantly greater than the reports of up to 15 kg N ha^-1^ yr^-1^ in Western Europe and United States [53]. Even though the estimated nutrient return from fine root turnover or deposition was lower than that from aboveground litterfall, their amounts were apparently unignorable for assessing nutrient cycling and nutrient supply, and for consequent evaluation of system sustainable, in poplar plantations.

Compared with the corresponding values reported for Chinese fir plantations, the most important plantation in southern China, poplar showed lower annual nutrient uptakes of N, P, K in the aboveground part, but a higher annual nutrient return. Also, the cycling coefficients of N, P, K, Ca and Mg were higher in poplar plantations than those in Chinese fir plantations (Table 4), indicating that less nutrients are retained in poplar than in Chinese fir. Nutrient removal via harvesting may exceed nutrient inputs in plantation systems [12, 22], while it may minimize nutrient removals via harvesting for those tree species with high nutrient cycling coefficients in some cases. Some studies have reported that nutrient cycling coefficients increase gradually with stand age [23, 25, 36, 54]. As mentioned above, the 10-year-old *P*. *deltoides* plantation was not yet at the optimal stage for harvest (Fig 2). Therefore, we speculate that the nutrient cycling coefficient would increase if the harvest rotation was extended for poplar plantations. This would minimize soil nutrient depletion. The proportions of nutrient-rich tissues in total tree biomass decreased with increasing stand age, as discussed above. Thus, appropriately extending the harvest rotation would benefit the long-term sustainability of the plantation system.

## Conclusions

Of the tree parts evaluated in the 10-year-old poplar plantation, stemwood showed the largest biomass, but relatively low nutrient content. The NUEs of poplar stemwood for N, P and K were greater than those of other comparable plantation trees in southern China. When compared with the corresponding values reported for Chinese fir plantations, the most important plantation tree species in southern China, poplar plantation showed higher cycling coefficients of N, P, K, Ca, and Mg. Therefore, to prevent nutrient losses and soil degradation, we recommend extending the harvest rotation, and changing from whole-tree harvesting to stemwood-only harvesting, which reserve other tissues with high nutrient contents on site. These strategies are expected to be able to minimize site deterioration. Continuous monitoring of the nutrient characteristics in poplar plantations is also necessary to support sustainable production in the future.
